# Reduced Number and Activity of Circulating Endothelial Progenitor Cells in Acute Aortic Dissection and Its Relationship With IL-6 and IL-17

**DOI:** 10.3389/fcvm.2021.628462

**Published:** 2021-03-29

**Authors:** Zhenhua Huang, Zhihao Liu, Keke Wang, Zi Ye, Yan Xiong, Bin Zhang, Jinli Liao, Lijing Zeng, Haitao Zeng, Gexiu Liu, Hong Zhan, Zhen Yang

**Affiliations:** ^1^Division of Emergency Medicine, Department of Emergency Intensive Care Unit, The First Affiliated Hospital of Sun Yat-sen University, Guangzhou, China; ^2^Department of Cardiovascular Disease, Jiangmen Central Hospital, Affiliated Jiangmen Hospital of Sun Yat-sen University, Jiangmen, China; ^3^Clinical Experimental Center, Jiangmen Central Hospital, Affiliated Jiangmen Hospital of Sun Yat-sen University, Jiangmen, China; ^4^Department of Reproductive Medicine, The Sixth Affiliated Hospital of Sun Yat-sen University, Guangzhou, China; ^5^School of Basic Medicine and Public Health Medicine, Institute for Hematology, Jinan University, Guangzhou, China

**Keywords:** acute aortic dissection, endothelial progenitor cells, endothelial injury, IL-6, IL-17

## Abstract

This study investigates the alteration in function and number of circulating endothelial progenitor cells (EPCs) in patients with aortic dissection (AD), compared with hypertensive patients, and its possible mechanism. Thirty-four patients with acute aortic dissection (AAD) and 20 patients with primary hypertension were involved. Flow cytometry analysis was performed to detect the number of CD34+/KDR+ cells, and acetylated low density lipoprotein (ac-LDL) and lectin fluorescent staining method was applied to test the number of cultured EPCs. In addition, EPC migration and proliferation were measured, and plasma interleukin 6 (IL-6) and interleukin 17 (IL-17) levels were investigated. The number of circulating EPCs in the AAD group was lower than that in the non-AD group, and the proliferation and migration of circulating EPCs in the AAD group were lower than that in the non-AD group. In addition, the number, proliferation, and migration of circulating EPCs were significantly inversely correlated with the aortic dissection detection risk score (ADD-RS). More importantly, increased plasma IL-6 and IL-17 level was found in the AAD group, and the two inflammatory factors were inversely associated with the function and number of circulating EPCs in the AAD group. We first demonstrated that the number and function of circulating EPCs are reduced in the AAD group, which may be partly related to upregulated plasma IL-6 and IL-17. Our study provides novel insight on the underlying mechanism and potential therapeutic target of AAD.

## Introduction

Aortic dissection (AD) is a serious disease threatening human life, which has rare incidence rate and extremely high mortality (1, 2). The incidence of acute aortic dissection (AAD) ranges from 3 to 6 per 100,000 patient-years in the general population in the United States, which has been increasing markedly, especially in recent years (3). It is well-known believed that the changes in the membrane structure and the function of critical cells in the aorta play an important role in the pathogenesis of AAD (4, 5). The aortic wall includes the intima, middle layer, and adventitia. The intimal rupture is one of the initial events of AD. Previous studies on the intimal layer of the aorta mainly concentrated in the pathogenesis of atherosclerotic stenosis such as coronary heart disease, and in recent years, the structure and function of the intimal layer of the aorta have been paid more attention (6, 7).

Aortic intima functions as critical factor to maintain the normal morphology and diastolic function of arteries (8). Some studies have shown that the damage and tear of vascular intima are the initial link of aortic dissection (9) and proved that the intima plays a crucial role in vascular injury and repair. Another study also have proven that vascular endothelial cell is damaged, promoting the formation of AD (10). Therefore, the repair of the intima may provide a novel way to intervene the pathological progress of aortic dissection.

Endothelial progenitor cells (EPCs) mobilized from bone marrow can directly differentiate into endothelial cells. They not only participate in the formation of early embryonic blood vessels but also participate in the regeneration of adult blood vessels (11, 12). EPCs are mainly involved in the development of the blood system and inflammatory immune response with inability for vascular regeneration, which can accelerate the repair in damaged vascular endothelium. EPCs have the characteristics of proliferation, migration, adhesion, and basement membrane matrix formation during the process of vascular repair (13, 14).

Many studies confirmed that metabolic abnormalities, smoking, and obesity can decrease circulating number and function of EPCs, reduce its ability to repair the endothelium, and cause endothelial dysfunction, which in turn give rise to the process of vascular diseases including hypertension (15–17). A systematic review summarized that 45–100% patients with acute aortic dissection have a most common comorbidity with hypertension (3). In our previous study, we found that the early stage of hypertension can cause EPC dysfunction (18, 19). Therefore, we speculate that endothelial injury may be the main cause of AAD.

Inflammation may be engaged in the development of AAD (20). During the process of AAD, inflammatory cells, such as phagocytes, can degrade elastic fibers by releasing matrix metalloproteinase (MMP), destroy the middle membrane of the aorta, and cause the thinning of the arterial wall, which leads to AD (21, 22). Interleukin 6 (IL-6) is an important proinflammatory factor, which can induce monocytes to differentiate into phagocytes (23). IL-6 was found to increase in acute aortic dissection, and when downregulating IL-6 expression, vascular macrophages production were decreased, thus delaying the occurrence of aortic dissection (24). Interleukin 17 (IL-17), a CD4+ T-helper subset, has been found to be involved in atherosclerosis and vascular dysfunction (25, 26). Previous study reported that IL-17 may function as a participant in AD pathogenesis by promoting inflammation (27). Thus, IL-6 and IL-17 may be related with AAD for its vascular inflammation.

This study is to explore the relationship between the function and number of EPC and aortic dissection detection risk score (ADD-RS) in AAD and hypertensive patients and analyze the level of IL-6 and IL-17 in the two groups. Our purpose is to find the potential molecular mechanism of AAD and a new intervention target for AAD prevention and treatment.

## Materials and Methods

### Subject Characteristics

Thirty-four patients suffering AAD, and 20 non-AD patients with essential hypertension were recruited. All patients older than 18 years admitted to the First Affiliated Hospital of Sun Yat-sen University were eligible. The definition of AD in our study was according to a previous study, which was majorly diagnosed by computed tomography (CT) scan (28). AAD was defined as any dissection that involved the aorta presenting within 14 days of symptom onset (28). The investigation protocol was approved by the Ethical Committee of the First Affiliated Hospital of Sun Yat-sen University. The basic characteristics of two groups are shown in Table 1.

**Table 1:** Clinical and biochemical characteristics in AAD and Non-AD groups.

**Characteristics**	**AAD group (*n* = 34)**	**Non-AD group (*n* = 20)**
Sex(M), *n* (%)	26 (76.5%)	13 (65%)
Age (years), mean(SD)	57.4 (10.4)	59.0 (10.9)
BMI (kg/cm^2^), mean(SD)	25.2 (3.4)	25.2 (2.9)
Heart rate (time/min), mean (SD)	77.0 (9.6)	77.1 (8.7)
Systolic blood pressure (mmHg), mean (SD)	161.4 (19.4)	166.7 (16.7)
Diastolic blood pressure (mmHg), mean (SD)	86.8 (12.1)	88.5 (18.5)
cTn-T (ug/L), mean(SD)	0.09 (0.24)	0.04 (0.1)
ALT (mmol/L), mean (SD)	93.4 (188.6)	28.3 (12.6)
AST (mmol/L), mean (SD)	78.9 (109.8)	25.1 (6.3)^*^
BUN (mmol/L), mean (SD)	8.3 (7.6)	6.9 (5.2)
Cr (mmol/L), mean(SD)	164.5 (232.9)	113.0 (134.8)
GLU (mmol/L), mean (SD)	7.0 (2.3)	6.3 (2.7)
CRP (mg/L), mean (SD)	66.4 (52.8)	5.2 (5.8)^*^
WBC (^*^10^9^/L), mean (SD)	12.5 (4.6)	7.9 (1.7)^*^
TC (mmol/L), mean (SD)	4.5 (1.0)	4.5 (1.2)
TG (mmol/L), mean (SD)	1.5 (0.6)	1.6 (0.9)
HDL (mmol/L), mean (SD)	1.0 (0.3)	1.1 (0.4)
LDL (mmol/L), mean (SD)	2.9 (0.8)	2.8 (0.8)
D-dimer (mg/L FEU), mean (SD)	15.2 (36.2)	0.4 (0.3)^*^
**Previous history**		
Smoker, *n* (%)	14 (41.2)	8 (40)
CAD, *n* (%)	4 (11.7)	3 (15)
Hypertension, *n* (%)	34 (100)	20 (100)
Hypercholesterolemia, *n* (%)	7 (20.6)	3 (15)
Diabetes, *n* (%)	6 (17.6)	3 (15)

*BMI, body mass index; cTn-T, Cardiac troponin-T; AST, aspartate amino transferals; ALT, alanine transaminase; BUN, blood urea nitrogen; Cr, serum creatinine; CRP, C-reactive protein; TC, total cholesterol; WBC, white blood cell; TG, triglyceride; HDL, high density lipoprotein; LDL, low-density lipoprotein*.

*Data are given as mean ± SD*.

* *P < 0.05 vs. Non-AD group*.

### Data Collection and ADD-RS Assessment

Data collection for the assessment of the pretest probability of AAD were performed by a medical researcher in the Emergency Department. The tool applied to evaluate the pretest probability of AAD was the ADD-RS. The ADD-RS of AAD patient was evaluated according to the previous study (29).

### The Evaluation of EPC Number and Activity

Circulating EPC number was detected by cell culture assay and flow cytometry analysis as previous studies (19, 30, 31). The EPC migration and proliferation assay was implemented according to previous studies (19, 30, 31).

### Measurement of Plasma Levels of IL-6 and IL-17

Plasma levels of IL-6 and IL-17 were assessed as previously described (32, 33). According to the instructions of the manufacturer, the human IL-6, and IL-17 levels in plasma were determined by human IL-6 and IL-17 Quantikine ELISA kit (R&D Systems, Minneapolis, USA).

### Statistical Analysis

All statistical analyses were carried out with SPSS 22.0. Continuous variables are represented by mean ± SD, and the comparison between two groups was analyzed by Student's *t*-test. Categorical variables were compared using chi-squared analysis. Pearson's coefficient (*r*) was used to calculate univariate correlations. If a null hypothesis could be rejected at *p* < 0.05, statistical significance was assumed.

## Results

### Baseline Characteristics

As Table 1 shows, the study population was predominantly male. The two groups were similar in terms of age and sex. The aspartate aminotransferase (AST), white blood cell (WBC), C-reactive protein (CRP), and D-dimer levels were significantly higher than those in the non-AD group (*p* < 0.05). There were no differences in the levels of heart rate, diastolic blood pressure, systolic blood pressure, body mass index (BMI), alanine aminotransferase (ALT), low-density lipoprotein (LDL), total cholesterol (TC), high-density lipoprotein (HDL), triglycerides (TG), blood urea nitrogen (BUN), creatinine (Cr), and cardiac troponin T (cTn-T) in the two groups (*p* > 0.05). In addition, according to the previous investigation for the International Registry of Acute Aortic Dissection (IRAD) Classification System to characterize survival after aortic dissection (34), the number of hyperacute patients is 19, the number of acute patients is 9, and the number of subacute patients is 6.

### Circulating EPC Number and Function in AD and Non-AD Groups

**Figure 1:**
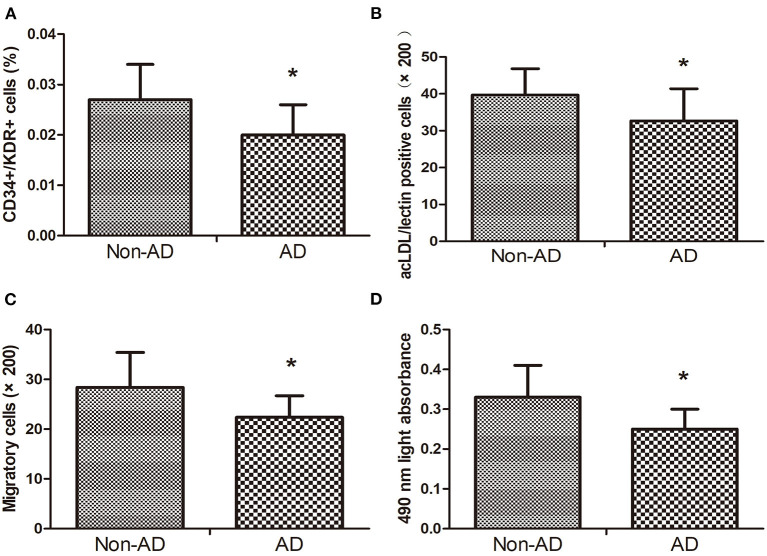
The CD34+KDR+ positive cells **(A)** and DiI-acLDL/lectin positive cells **(B)** in AD subjects were lower than those in Non-AD subjects. The migratory **(C)** and proliferative **(D)** activities of EPCs in AD subjects decreased when compared with Non-AD subjects. Data are given as the mean ± standard deviation (SD). *p < 0 05 vs. Non-AD group.

Compared with the non-AD group, circulating EPC number detected by CD34+/KDR+ and DiI-acLDL/lectin double-positive cells in AAD group was reduced (Figures 1A,B). Moreover, compared with the non-AD group, EPC migration and proliferation in the AAD group were remarkably declined (Figures 1C,D).

### EPC Number and Function in Low and High ADD-RS

**Figure 2:**
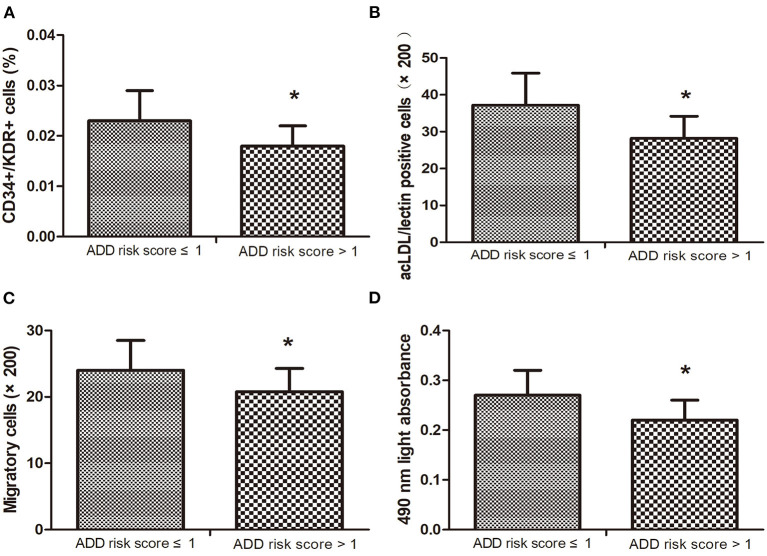
The number of circulating endothelial progenitor cells (EPCs) **(A,B)** in high ADD-RS group were lower than that in low ADD-RS group. The migratory **(C)** and proliferative **(D)** activities in low ADD-RS group were higher than that in high ADD-RS group. Data are given as the means ± standard deviation (SD). *p < 0 05 vs. ADD risk score ≤ 1 group.

According to the previous report (29), the ADD-RS is categorized as low ADD-RS group if ADD-RS ≤ 1 (defining low clinical probability of AAD) and high ADD-RS group if ADD-RS > 1 (defining high clinical probability of AAD). In our data, the number of patients with ADD-RS ≤ 1 and ADD-RS > 1 are both 17. Interestingly, the EPC number and function were found to decrease in high ADD-RS group, compared with the low ADD-RS group (Figures 2A–D).

### Relationship Between Characteristics of Circulating EPCs and ADD-RS

**Figure 3:**
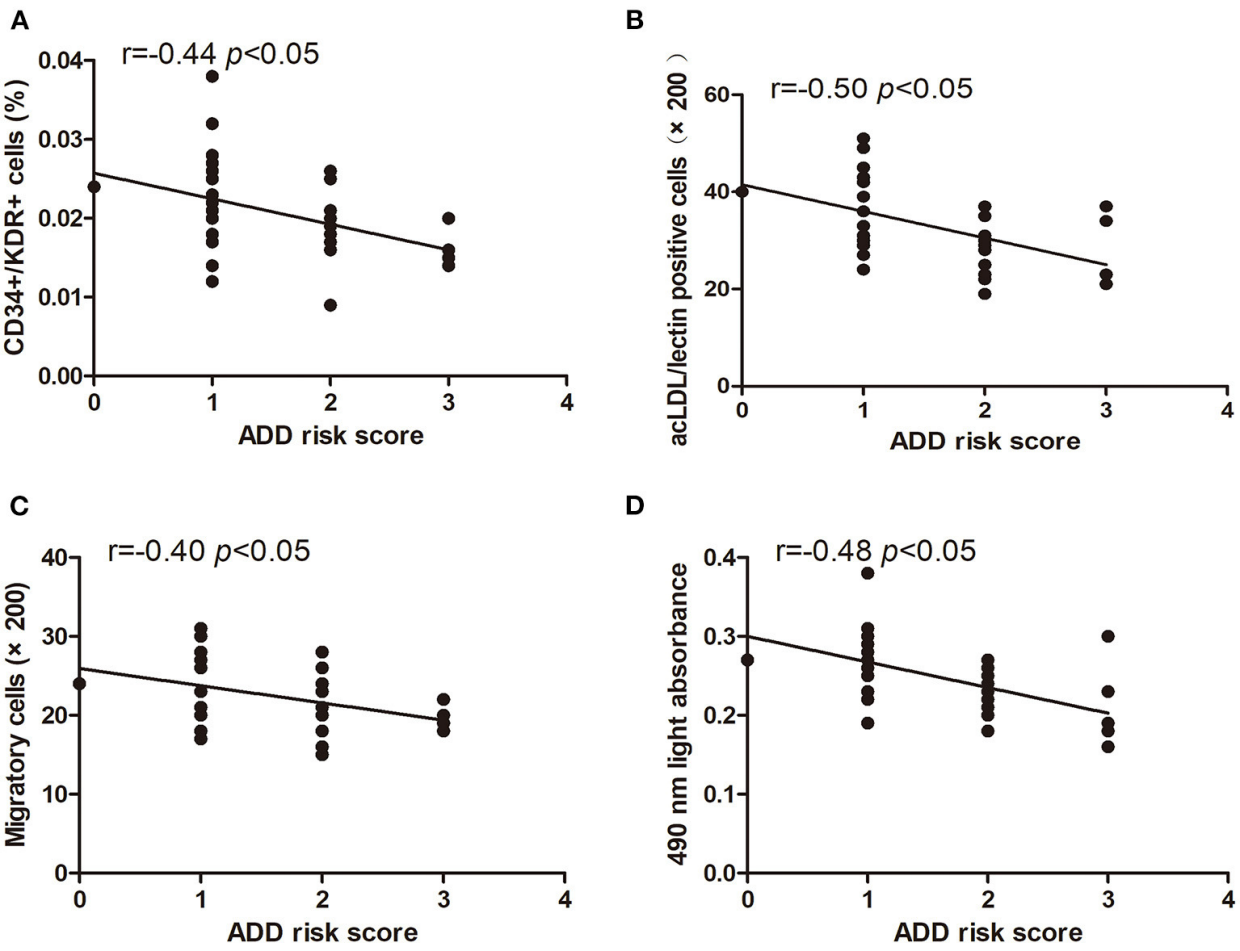
Pearson correlation ratios were used to analyze the relationship between the number or function of EPCs and ADD-RS. The CD34+KDR+ positive cells **(A)** and DiI-acLDL/lectin positive cells **(B)** significantly correlated with ADD-RS. The migratory **(C)** and proliferative **(D)** activities of EPCs were related to ADD-RS.

The levels of circulating CD34+/KDR+ cells (*r* = −0.44, *p* < 0.05) and DiI-acLDL/lectin double-positive cells (*r* = −0.50, *p* < 0.05) were inversely correlated with the score of ADD-RS (Figures 3A,B). In addition, we also found that the EPC migration and proliferation were inversely related to the score of ADD-RS (*r* = −0.40, *p* < 0.05, and *r* = −0.48, *p* < 0.05, respectively) (Figures 3C,D).

### Plasma IL-6 and IL-17 Levels in the Two Groups

**Figure 4:**
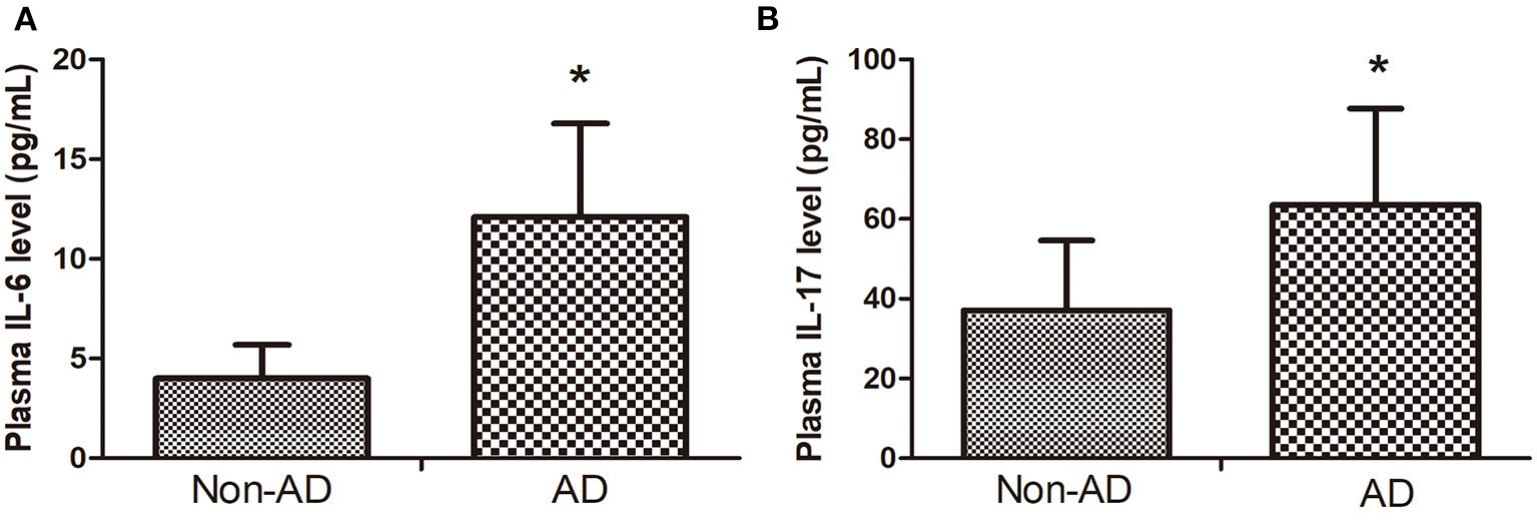
**(A)** The plasma IL-6 level in AAD group was higher than that in Non-AD group. **(B)** plasma IL-17 level in AAD group was higher than that in Non-AD group. Data are given as mean ± SD. *p < 0.05 vs. Non-AD group.

The plasma IL-6 and IL-17 level in the AAD group were higher than that in the non-AD group (Figures 4A,B).

### Relationship Between Characteristics of Circulating EPCs and IL-6 Level

**Figure 5:**
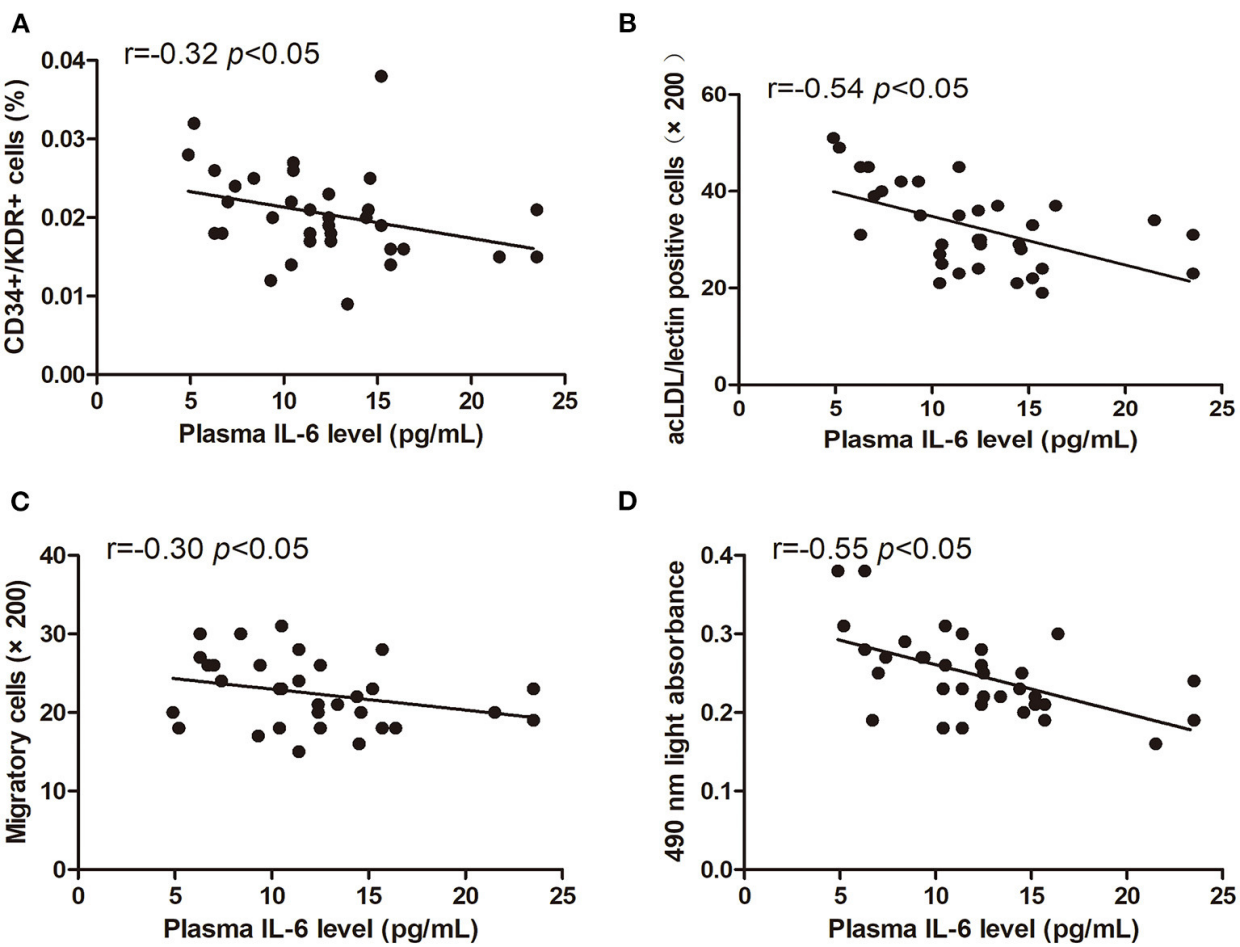
Pearson correlation ratios were used to analyze the relationship between the number or function of EPCs and plasma IL-6 level. The CD34+KDR+ positive cells **(A)** and DiI-acLDL/lectin positive cells **(B)** significantly correlated with IL-6. The migratory **(C)** and proliferative **(D)** activities of EPCs were related to IL-6.

The levels of circulating CD34+/KDR+ cells (*r* = −0.32, *p* < 0.05) and DiI-acLDL/lectin double-positive cells (*r* = −0.54, *p* < 0.05) were inversely correlated with the plasma IL-6 level (Figures 5A,B). Additionally, the EPC migration and proliferation were inversely related to the plasma IL-6 level (*r* = −0.30, *p* < 0.05, and *r* = −0.55, *p* < 0.05, respectively) (Figures 5C,D).

### Relationship Between Characteristics of Circulating EPCs and IL-17 Level

**Figure 6:**
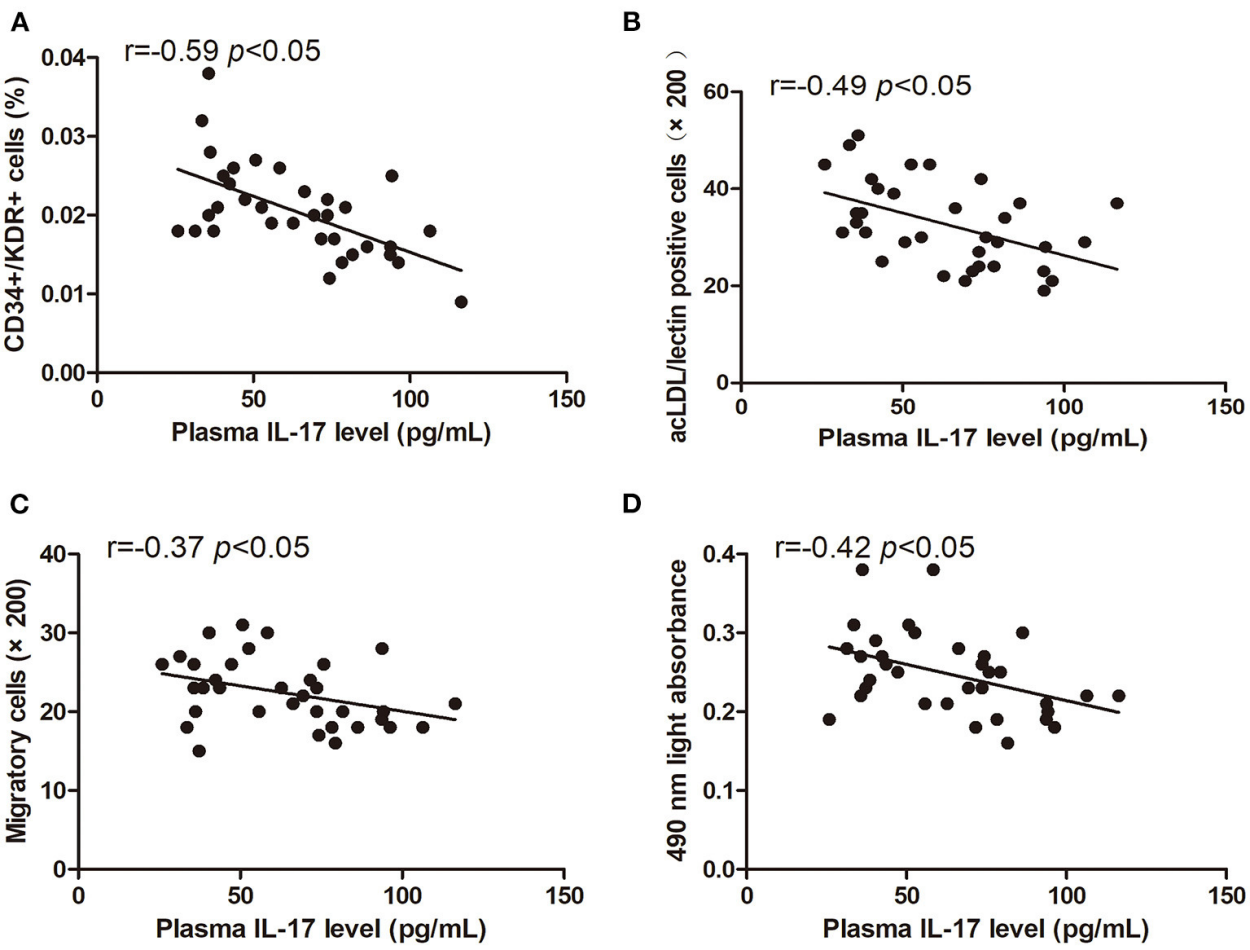
Pearson correlation ratios were used to analyze the relationship between the number or function of EPCs and plasma IL-17 level. The CD34+KDR+ positive cells **(A)** and DiI-acLDL/lectin positive cells **(B)** significantly correlated with IL-17. The migratory **(C)** and proliferative **(D)** activities of EPCs were related to IL-17.

The levels of circulating CD34+/KDR+ cells (*r* = −0.59, *p* < 0.05) and DiI-acLDL/lectin double-positive cells (*r* = −0.49, *p* < 0.05) were inversely correlated with the plasma IL-17 level (Figures 6A,B). The EPC migration and proliferation were inversely related to the plasma IL-17 level (*r* = −0.37, *p* < 0.05, and *r* = −0.42, *p* < 0.05, respectively) (Figures 6C,D).

## Discussion

Our study has first demonstrated that EPC number, migration, and proliferation in acute aortic dissection patients are significantly reduced. We further revealed that there is an inversely relationship between the circulating EPCs and the severity of AAD evaluated by ADD-RS exists. Moreover, the results indicated that the plasma IL-6 and IL-17 levels are increased in AAD patients and negatively correlated with EPC number or function. Taken together, the proinflammatory factor may be partly associated with the attenuated endogenous endothelial repair ability and participate in the process of AAD.

Numerous studies have indicated that the EPCs may predict outcomes in cardiovascular disease (35, 36). It has been proven that the levels of CD34+ cells are altered in patients with abdominal aortic aneurysms compared with patients with peripheral vascular disease, and the CD34+ cells were correlated with aneurysm diameter (37, 38). However, few studies focus on the relationship between circulating EPCs and AAD.

In the current study, circulating EPC number or function was investigated. All patients with acute aortic dissection involved in our study have hypertension. Our previous study discovered a dysfunction of circulating EPCs in hypertension (12, 19). Hereby, the hypertensive patients were recruited in the non-AD group to eliminate the influence of blood pressure on circulating EPCs. The results showed that AAD patients had decreased number and migration or proliferation of EPCs. However, our findings may be the different from that of Van Spyk E N's (37), and the possible reasons may be as follows: First, there is a difference in enrolled subjects. although commonly pathogenesis of AAD is atherosclerosis and share similar as the abdominal aortic aneurysm (AAA). However, vascular injury of AAD patients enrolled in our study may be more serious than that of AAA enrolled in the previous investigation. Second, we used CD34+KDR+ cells defined as EPCs, which is a frequently used and credible method to evaluate putative antigenic phenotypes of EPCs. However, the surface antigen CD34+ cells in Van Spyk E N's study, as a hematopoietic multilineage stem cell, are able to differentiate into myeloid, lymphoid, erythroid, and megakaryocytic cells (39). Third, in our study, EPCs were isolated from the peripheral blood mononuclear cells, and its migration and proliferation were also investigated.

ADD-RS may provide a simple and systematic method to screen the high likelihood of patients with AAD at the Emergency Department (ED) (40). On the basis of the ADD-RS, patients can be classified into two categories (ADD-RS ≤ 1, ADD-RS >1), which are adopted as diagnostic algorithms by international guidelines for AAD (29, 41). EPCs have been proven to be a predictive factor for cardiovascular diseases (42, 43). We revealed that EPC number or function were lower in high ADD-RS group than that in low ADD-RS group. Moreover, there is a remarkable inverse association between EPC number or function and ADD-RS. These results manifest that circulating EPCs may act as a surrogate indicator to evaluate the probability of AAD occurrence with the development of vascular diseases. In our study, the AAD patient in acute phase is defined according to a previous investigation (28). However, according to the previous investigation for the IRAD Classification System to characterize survival after aortic dissection (34), most patients are in the hyperacute or acute phase, and few patients are in the subacute phase. Our further study will be performed to investigate the effect of different classification of AD on the related alteration in circulating EPCs.

The development of AAD is proved to be accompanied by inflammation (44). However, it is not clear whether inflammation contributes to regulating the circulating EPCs in AAD. Previous studies have demonstrated that IL-6 and IL-17 take part in the mechanism of AAD (24, 27). Interestingly, our results found that IL-6 or IL-17 level had a negative correlation with EPC number and migration or proliferation in AAD, indicating the possible relationship between the proinflammatory factor and the circulating EPCs in AAD. It was reported that IL-6 has an unfavorable effect on the function of EPC after myocardial infarction (44), but it is unknown whether IL-17 can regulate the circulating EPCs. As we all know, IL-17 cytokine, produced by a novel subset of CD4+ helper T cell, has a critical effect in chronic inflammatory diseases and AAD (45) and was found to activate some common downstream signaling pathway, such as tumor necrosis factor-α (TNF-α), IL-6, and IL-1(46). The current study revealed that inflammatory-mediated endothelial repair capacity incompetency may be implicated in AAD development.

The current study has some limitations. First, this study has relatively small sample size. Further large-sample research needs to be carried out to further address it. Second, our study is designed to eliminate the influence of blood pressure on EPCs, but acute inflammation or other inflammatory disease also has an effect on EPC number and function. Our further study will be performed to elucidate it. Finally, the data of EPCs before the occurrence of AAD are difficult to acquire and not presented in this study. We will further compare the quantitative and qualitative alteration in EPCs before and after AAD in animal experiment.

In summary, our findings have some clinical implications. First, circulating EPCs may be a novel surrogate biomarker for predicting the probability of AAD. Second, the improved function of EPCs can enhance endogenous endothelial repair capacity, which may be beneficial to the prevention and treatment of AAD. For example, ACEI and ARB, significantly increasing function of EPCs, may prevent the occurrence of AAD. Third, proinflammatory factor, including IL-17 and IL-6, is involved in attenuated endogenous endothelial repair ability and may be engaged in the development of AAD. It suggests that anti-inflammatory agents such as statis and PCSK9 may be a new strategy for preventing and treating AAD.

## Data Availability Statement

The raw data supporting the conclusions of this article will be made available by the authors, without undue reservation, if it is permitted by all authors.

## Ethics Statement

The studies involving human participants were reviewed and approved by the Ethical Committee of the First Affiliated Hospital of Sun Yat-sen University. The patients/participants provided their written informed consent to participate in this study.

## Author Contributions

HZh and ZYa designed the research. ZH, ZL, KW, ZYe, BZ, YX, JL, LZ, HZe, and GL performed the research. ZH, ZL, KW, HZh, and ZYa analyzed the data. ZH, ZL, and ZYa wrote the paper. All authors contributed to the article and approved the submitted version.

## Conflict of Interest

The authors declare that the research was conducted in the absence of any commercial or financial relationships that could be construed as a potential conflict of interest.
